# Quality of life before intensive care unit admission is a predictor of survival

**DOI:** 10.1186/cc5970

**Published:** 2007-07-13

**Authors:** José GM Hofhuis, Peter E Spronk, Henk F van Stel, Augustinus JP Schrijvers, Jan Bakker

**Affiliations:** 1 Department of Intensive Care Medicine, Gelre Hospitals (location Lukas), Albert Schweitzerlaan, 7334 DZ Apeldoorn, The Netherlands; 2 Department of Intensive Care Medicine, Erasmus Medical Centre, Gravendijkwal 230, Rotterdam, 3015 CE, The Netherlands; 3 Julius Center for Health Sciences and Primary Care, University Medical Centre Utrecht, Heidelberglaan 100, Utrecht, 3584 CX, The Netherlands; 4 Department of Medical Decision Making, Leiden University Medical Centre, Albinusdreef 2, Leiden, 2333 ZA, The Netherlands

## Abstract

**Introduction:**

Predicting whether a critically ill patient will survive intensive care treatment remains difficult. The advantages of a validated strategy to identify those patients who will not benefit from intensive care unit (ICU) treatment are evident. Providing critical care treatment to patients who will ultimately die in the ICU is accompanied by an enormous emotional and physical burden for both patients and their relatives. The purpose of the present study was to examine whether health-related quality of life (HRQOL) before admission to the ICU can be used as a predictor of mortality.

**Methods:**

We conducted a prospective cohort study in a university-affiliated teaching hospital. Patients admitted to the ICU for longer than 48 hours were included. Close relatives completed the Short-form 36 (SF-36) within the first 48 hours of admission to assess pre-admission HRQOL of the patient. Mortality was evaluated from ICU admittance until 6 months after ICU discharge. Logistic regression and receiver operating characteristic analyses were used to assess the predictive value for mortality using five models: the first question of the SF-36 on general health (model A); HRQOL measured using the physical component score (PCS) and mental component score (MCS) of the SF-36 (model B); the Acute Physiology and Chronic Health Evaluation (APACHE) II score (an accepted mortality prediction model in ICU patients; model C); general health and APACHE II score (model D); and PCS, MCS and APACHE II score (model E). Classification tables were used to assess the sensitivity, specificity, positive and negative predictive values, and likelihood ratios.

**Results:**

A total of 451 patients were included within 48 hours of admission to the ICU. At 6 months of follow up, 159 patients had died and 40 patients were lost to follow up. When the general health item was used as an estimate of HRQOL, area under the curve for model A (0.719) was comparable to that of model C (0.721) and slightly better than that of model D (0.760). When PCS and MCS were used, the area under the curve for model B (0.736) was comparable to that of model C (0.721) and slightly better than that of model E (0.768). When using the general health item, the sensitivity and specificity in model D (sensitivity 0.52 and specificity 0.81) were similar to those in model A (0.45 and 0.80). Similar results were found when using the MCS and PCS.

**Conclusion:**

This study shows that the pre-admission HRQOL measured with either the one-item general health question or the complete SF-36 is as good at predicting survival/mortality in ICU patients as the APACHE II score. The value of these measures in clinical practice is limited, although it seems sensible to incorporate assessment of HRQOL into the many variables considered when deciding whether a patient should be admitted to the ICU.

## Introduction

It is difficult for doctors to predict whether a critically ill patient will survive intensive care treatment. Mortality in patients admitted to intensive care units (ICU) remains high [1]. An increasing number of in-hospital patients die in the ICU [2]. The advantages of a validated strategy to identify those patients who will not benefit from ICU treatment are evident. Providing critical care treatment to patients who will ultimately die in the ICU is accompanied by an enormous emotional and physical burden for both patients and their relatives. Furthermore, ICU resources are scarce, and identifying those patients who will not survive intensive care treatment allows us to make better use of what resources are available [3]. The available predicting tools, including the Acute Physiology and Chronic Health Evaluation (APACHE) II score, are based on a combination of pre-morbid factors and acute physiology items recorded during the first 24 hours after admission. The use of these systems in individual patients is limited because they have been validated at the group level. Consequently, ICU doctors must rely upon their clinical experience in their decision making. The predictive value of clinical experience in this regard is also limited [4]. We hypothesized that the perceived health-related quality of life (HRQOL) of patients also reflects components of 'physiological reserve' and could, as such, act as a predictor of mortality.

The goal of the present study was to evaluate the predictive value for survival of the pre-admission HRQOL, alone and in combination with the APACHE II score, in critically ill patients.

## Materials and methods

**Figure 1:**
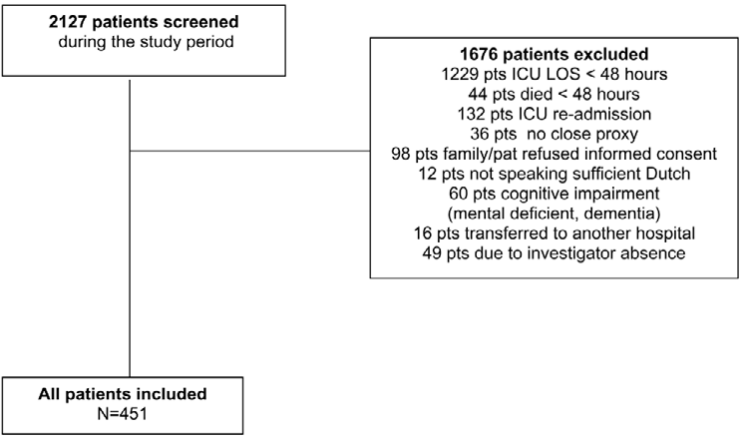
Flow diagram of patient selection and inclusion. Follow up was lost in 40 patients, usually because the patients did not live in the area of the hospital (they were on vacation). Characteristics of those patients did not differ from those of the group analyzed in the study (data not shown). A large group of patients (*n *= 1,229) were admitted to the intensive care unit (ICU) for under 48 hours and hence were excluded from the final analysis. Patients who died within 48 hours of ICU admission (*n *= 44) were excluded. In some cases the patient had no close proxy (*n *= 36). Patients re-admitted to the ICU were excluded (*n *= 132) because it was possible that the first admission could have biased the proxy memories of the patient's pre-admission health-related quality of life (HRQOL). Proxies or the patients themselves refused informed consent (*n *= 98) mainly because they felt study participation to be too great a burden at that stressful moment. Patients transferred to other hospitals (*n *= 16) or with cognitive impairment (*n *= 60), or who did not speak sufficient Dutch (*n *= 12) were also excluded. Some patients were not included because of investigator absence (*n *= 49). LOS, length of stay.

All patients admitted for more than 48 hours to the 10-bed mixed surgical-medical ICU of the Gelre Lukas hospital in Apeldoorn (a 654-bed, university-affiliated hospital in The Netherlands) were eligible for the study. We included only patients with a ICU stay of longer than 48 hours because we aimed to evaluate the sickest patients, hypothesizing that those patients were more likely to die. We felt that proxies of patients who would die during the first 48 hours after ICU admission should not be burdened with study participation. Between September 2000 and April 2004, all admitted patients were screened for eligibility for study participation (Figure 1). The local ethics committee approved the study. Informed consent was given by a close relative and as soon as possible by the patient. Mortality was evaluated from ICU admittance until 6 months after ICU discharge. The severity of illness was routinely measured using the APACHE II score [5]. Physicians treating the patients were not aware of the pre-admission HRQOL.

### Health-related quality of life measurement

The Short-form 36 (SF-36, version 1; ^© ^1993 Medical Outcome Trust), a generic, widely used standardized health status questionnaire, was used to measure HRQOL. This measurement contains eight multi-item dimensions: physical functioning, role limitation due to physical problems, bodily pain, general health, vitality, social functioning, role limitation due to emotional problems, and mental health. Answers to the 36 items were transformed, weighed and subsequently scored according to predefined guidelines [6]. Higher scores represent better functioning, with a range from 0 to 100. Furthermore, scores were aggregated to summary measures representing a physical component score (PCS; mainly reflecting physical functioning) and a mental component score (MCS; mainly reflecting social functioning and mental health) [7]. Population scores on PCS and MCS have been standardized on 50 as population mean (SD 10 representing 1) [7]. For the PCS, very high scores indicate no physical limitations, disabilities, or decrements in well being, as well as high energy levels. Very low scores indicate substantial limitations in self-care and in physical, social and role activities, severe bodily pain, or frequent tiredness [7]. For the MCS, very high scores indicate frequent positive effect, absence of psychological distress, and limitations in usual social/role activities caused by emotional problems. Very low scores indicate frequent psychological distress, and substantial social and role disability due to emotional problems [7].

Translation, validation and generating normative data of the Dutch language version of the SF-36 health questionnaire were evaluated in 1998 in community and chronic disease populations [8]. Because most of the patients in our study were unable to complete a questionnaire at the time of admission, proxies had to be used as a surrogate approach. In proxies and patients the same method was used to complete the SF-36. The use of proxies to assess the patients' HRQOL using the SF-36 in the ICU setting was validated in earlier studies conducted by our group [9] and others [10,11]. HRQOL was measured within 48 hours of ICU admission (estimation of HRQOL up to 4 weeks before admission). All interviews were performed by the same investigator (JH). The average time required to complete the questionnaire was 15 to 20 min. Consideration of multiple items has the advantage of allowing construction of a comprehensive profile of HRQOL, but it may burden the critically ill patient. We used the first question of the SF-36 as a primary approach to estimation of the patient's HRQOL. This is the single-item question pertaining general health status; 'In general, would you say your health is excellent, very good, good, fair, or poor?' [12,13]. The advantages of such a single-item question are its simplicity and ease of application.

### Statistical analysis

A Pearson's χ^2 ^test was used to assess demographic differences between ICU survivors and ICU non survivors. The differences between scores for the single-item question were tested using the χ^2 ^test for trend. We examined the relationship between the single-item question on HRQOL before ICU admission and mortality at 6 months after ICU discharge with multivariate logistic regression using the variables known on the first day of ICU admission (APACHE II score), adjusted for age and sex.

To analyze the potential of variables to predict mortality in patient subgroups, we used five statistical models. HRQOL was entered as the response to the single-item question, or as MCS and PCS. In the model A we included the general health item of the SF-36, age and sex. In model B we included both the PCS and MCS from the SF-36, and age and sex. In model C we included APACHE II score, age and sex. In model D we included the general health item of the SF-36, APACHE II score, age and sex. In model E we included both the PCS and MCS from the SF-36, APACHE-II score, age and sex.

To estimate the ability to discriminate between survivors and non-survivors, odds ratios were calculated, receiver operating characteristic analysis was performed and the area under the curve (AUC) was calculated. Classification tables were used to assess the sensitivity for observed deaths being labeled by the models as predicted deaths, specificity for a predicted death being an observed death, and positive and negative predictive values and likelihood ratio. Data were analyzed using SPSS (version 11.5; SPSS Inc., Chicago, IL, USA). All data are expressed as median (interquartile range), unless indicated otherwise.

*P *< 0.05 was considered statistically significant.

## Results

During the study period, 451 patients (61.2% male and 38.8% female) were included. At 6 months after ICU discharge, 159 patients had died. Forty patients were lost to follow up (Figure 1). Demographic and clinical characteristics are shown in Table 1.

**Table 1:** Demographic and clinical characteristics

Characteristic	Included patients (*n *= 451)
Age (years)^a^	71.0 (63 to 71)
Sex (male/female; %)	61.2/38.8
APACHE II score^a^	19.0 (15 to 23)
ICU length of stay (days)^a^	8.0 (5 to 16)
Hospital length of stay (days)^a^	23.0 (14 to 40)
Ventilation days+	6.0 (3 to 13)
Type of admission (%)	
Nonsurgical^b^	53.2
Elective surgery^c^	8.7
Acute surgery^d^	38.1

^a^Median (interquartile range). ^b^All admissions other than surgical. ^c^Intensive care unit (ICU) admission was planned within a 24-hour period before surgery. ^d^Unplanned surgery. APACHE-II, Acute Physiology and Chronic Health Evaluation.

Of the 451 included patients, in a small proportion of patients (*n *= 23) pre-admission HRQOL was derived from the patients themselves, whereas all other SF-36 scores were obtained from proxies.

### Prediction models

**Figure 2:**
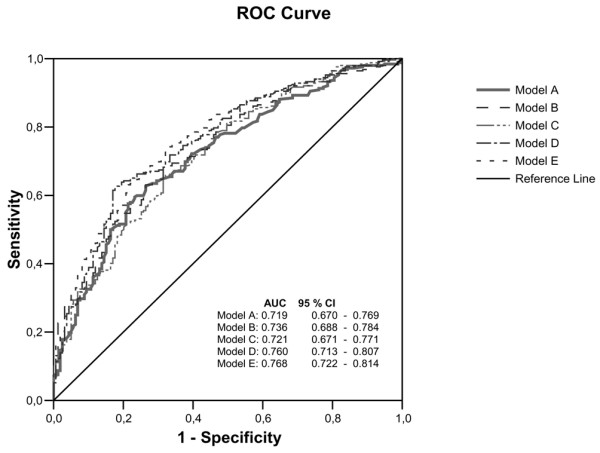
Receiver operating characteristic analysis of pre-admission HRQOL and APACHE II scores in relation to mortality. A total of 451 critically ill patients were included in the analysis. Model A included the general health item of the 36-item Short-form (SF-36), age and sex. Model B included the physical component score (PCS), mental component score (MCS), age and sex. Model C included the Acute Physiology and Chronic Health Evaluation (APACHE) II score, age and sex. Model D included the general health item of the SF-36, APACHE II score, age and sex. Model E included PCS, MCS, APACHE II score, age and sex. CI, confidence interval; HRQOL, health-related quality of life; ROC, receiver operating characteristic.

Using the single-item question on HRQOL as a potential predictor of survival, the AUC for model A (0.719) was comparable to that for the APACHE II score (model C; 0.721) and slightly better than that in model D (AUC = 0.760), in which both factors were combined (Table 2 and Figure 2). Comparable results were obtained when calculating odds ratios (Table 3) and with analysis using MCS and PCS in models B and E. The sensitivity and specificity in model D (sensitivity 0.52 and specificity 0.81) were similar to those in model A (0.45 and 0.80). Similar results were found when using PCS and MCS. In ICU patients (*n *= 451), sensitivity improved from 0.44 (model C; APACHE II score only) to 0.56 (model E; APACHE II score, and PCS and MCS), respectively. Results for specificity were similar, improving from 0.84 (model C; APACHE II score only) to 0.82 (model E; APACHE II score, and PCS and MCS). Similar results were also found when using the general health item (models A and D; Table 2). The negative and positive predictive values and likelihood ratios are shown in Table 2.

**Table 2:** Statistical characteristics of mortality prediction models in ICU patients

Characteristic	Model A	Model B	Model C	Model D	Model E
Sensitivity	0.45	0.50	0.44	0.52	0.56
Specificity	0.80	0.81	0.84	0.81	0.82
PPV	0.58	0.62	0.63	0.63	0.66
NPV	0.70	0.72	0.70	0.73	0.75
AUC	0.719	0.736	0.721	0.760	0.768
LR + (95% CI)	2.24 (1.66 to 3.02)	2.59 (1.93 to 3.48)	2.71 (1.95 to 3.77)	2.69 (2.00 to 3.60)	3.07 (2.28 to 4.12)
LR - (95% CI)	0.69 (0.59 to 0.80)	0.62 (0.52 to 0.73)	0.67 (0.58–0.78)	0.59 (0.50 to 0.71)	0.54 (0.45 to 0.65)

Model A included the general health item of the 36-item Short-form (SF-36), age and sex. Model B included the physical component score (PCS), mental component score (MCS), age and sex. Model C included the Acute Physiology and Chronic Health Evaluation (APACHE) II score, age and sex. Model D included the general health item of the SF-36, APACHE II score, age and sex. Model E included PCS, MCS, APACHE II score, age and sex. AUC, area under the curve; CI, confidence interval; HRQOL, health-related quality of life; ICU, intensive care unit; LR, likelihood ratio (+positive, -negative); NPV, negative predictive value; PPV, positive predictive value.

**Table 3:** Logistic regression models: odd ratios with 95% confidence intervals

	OR	95% CI	*P *value
Model A			

Sex	1.61	1.03 to 2.52	0.037
Age	1.06	1.04 to 1.09	<0.001
GH^a^	0.62	0.49 to 0.77	<0.001

Model B			

Sex	1.69	1.07 to 2.68	0.026
Age	1.07	1.04 to 1.09	<0.001
PCS	0.97	0.95 to 0.99	<0.001
MCS	0.96	0.94 to 0.98	<0.001

Model C			

Sex	1.74	1.11 to 2.74	0.016
Age	1.06	1.04 to 1.09	<0.001
APACHE II	0.09	1.05 to 1.13	<0.001

Model D			

Sex	1.80	1.13 to 2.86	0.013
Age	1.06	1.04 to 1.09	<0.001
GH^a^	0.60	0.48 to 0.76	<0.001
APACHE II	1.09	1.06 to 1.14	<0.001

Model E			

Sex	1.89	1.17 to 3.05	0.009
Age	1.06	1.04 to 1.09	<0.001
PCS	0.97	0.95 to 0.99	<0.001
MCS	0.96	0.94 to 0.98	0.001
APACHE II	1.09	1.05 to 1.13	<0.001

^a^General Health (GH) is item 1 from the SF-36: range 1 (poor) to 5 (excellent). The ranges for PCS and MCS are both 0 to 100. Model A included the general health item of the 36-item Short-form (SF-36), age and sex. Model B included the physical component score (PCS), mental component score (MCS), age and sex. Model C included the Acute Physiology and Chronic Health Evaluation (APACHE) II score, age and sex. Model D included the general health item of the SF-36, APACHE II score, age and sex. Model E included PCS, MCS, APACHE II score, age and sex. CI, confidence interval; OR, odds ratio.

The scores on the single-item question pertaining to general health status before ICU admission were higher in survivors than in the patients who died (*P *< 0.001), with respect to all, that is: excellent (3.6% of survivors versus 1.9% of those who died), very good (5.6% versus 4.4%), good (41.3% versus 18.9%), fair (38.1% versus 50.9%), or poor (11.5% versus 23.9%). Other possibly relevant variables such as the presence of severe sepsis, length of ICU and hospital stay, and ventilation days were included in the logistic regression analysis. However, because these variables did not contribute significantly to the prediction models, they were omitted from the final models, as described above.

## Discussion

We demonstrated that HRQOL before ICU admission can be used as a predictor of mortality in patients admitted to the ICU for longer than 48 hours. The mortality prediction ability of the pre-admission HRQOL estimated from the single-item question on the SF-36 was equal to those of the SF-36 (PCS and MCS) and the APACHE II score. Incorporating HRQOL into prediction models does not improve the predictive capacity of established models such as APACHE II and is not useful in clinical practice for making decisions in individual cases.

Mortality is difficult to predict for an individual patient because many factors determine survival from critical illness, such as age, sex, acute physiological deterioration and underlying illnesses. Several scoring systems aimed at predicting mortality have been developed that incorporate these factors. The APACHE II and III scores [5,14]., the Mortality Probability Model [15] and the Simplified Acute Physiology Score II [16] are established examples. When these systems were compared [17] their predictive ability, as judged by the AUC of the receiver operating characteristic curve, was around 70%, which is comparable to our findings. However, these scoring systems are only available after 24 hours of ICU admission, and they are highly specific (able to predict survival [specificity 90%]) but not very sensitive (less accurate in predicting death [sensitivity 50% to 70%]) [4].

The advantages of using pre-admission HRQOL as a predictor of mortality are that it is easily obtained and available as soon as the patient, or a proxy (close family member), in the case of incapacity, can be questioned. In particular, a single item like the first question of the SF-36 is advantageous because of its simplicity and ease of administration in seriously ill patients. However, this benefit may be obtained at the cost of detail in the information provided. Multiple-item scoring systems such as the SF-36 have the advantage of providing a complete profile of HRQOL, although they are more laborious and carry the risk of asking potentially irrelevant questions [13]. These two types of items (multiple and single) could be used together in the clinical setting.

Can HRQOL be used as an indicator of final outcome? Several studies have addressed this question in dialysis patients [18-20], coronary artery bypass graft surgery patients [21], patients with congestive heart failure [22] and those with advanced colorectal cancer [23].

Currently, HRQOL surveys are rarely used in ICU clinical practice, and they predominantly address the impact that critical illness has on HRQOL after ICU survival. Only a few studies have focused on the association between pre-admission HRQOL and survival in critically ill patients [24-26]. Yinnon and coworkers [24] analyzed HRQOL in a 1-week period preceding ICU admission using the linear analogue self assessment (LASA) score. Mortality was higher in patients with lower LASA scores, indicating worse HRQOL, than in those with higher LASA scores, indicating a good HRQOL. However, the LASA was developed for application in cancer patients receiving chemotherapy, and it has not been validated for use in critically ill patients. In addition, the period of 1 week preceding ICU admission may be rather short to conduct an adequate evaluation of HRQOL pre-emptively.

More recently, Welsh and coworkers [25] found that baseline patient functional status, as assessed by care providers, is correlated with mortality after ICU admission. However, that study is hampered by several drawbacks. Although the investigators also focused on patients with an expected ICU stay longer than 48 hours, they included only 9% of all ICU patients, which may indicate at least some form of selection bias. In addition, it may be questionable to correlate HRQOL scores directly with APACHE II scores without making any attempt to correct for confounding by multivariate analysis. Also, hospital deaths were not included in their analysis, which makes it difficult to understand the relation between HRQOL before ICU admission and mortality during or after critical illness.

The most recent work on this issue is that reported by Rivera-Fernandez and coworkers [26], who demonstrated in a multicentre study that HRQOL before ICU admission is related to ICU mortality, but that it contributes little to the discriminatory ability of the APACHE III prediction model and has little influence on ICU resource utilization, as indicated by length of stay in the ICU or therapeutic interventions [26]. However, the cohort they evaluated is not comparable with our patients, because at least 25% of the patients were admitted with a cardiac diagnosis, probably because coronary care units also participated in the study. Consequently, the number of surgical patients was only 24%, which is much lower than in a general ICU. In addition, the APACHE III score was used and related to a self-developed HRQOL questionnaire. Despite the differences that exist between these previous reports and ours, their findings are generally in accordance with ours and indicate that estimation of HRQOL before ICU admission deserves more attention by those caring for critically ill patients.

We conducted a long-term prospective study, which is an important strength of the data presented. Nevertheless, several limitations of our study should be mentioned. First, potential selection bias might have been present, because the HRQOL assessment could have influenced the decision to admit a patient to the ICU. However, we do not believe that this factor is important because the research nurse conducting the study did not communicate HRQOL findings to attending ICU physicians. Second, the APACHE II system was intended to be used to predict in-hospital mortality, not long-term mortality at 6 months or even later. However, repeating the analysis when omitting those patients who died after hospital discharge did not alter the results.

A third limitation of our study was the necessary use of proxies to evaluate pre-admission HRQOL instead of a retrospective assessment at ICU discharge could also have hampered results. We believe that this approach did not affect the final results, in view of the findings of previous validation studies [9-11]. Moreover, the use of proxies appears to be sensible, because critical illness itself could have influenced patients' recollections of their pre-admission health status. However, other groups have raised concerns about proxy estimations of HRQOL in populations with greater disease severity [27]. The same study suggested that predictions of poor ICU outcome may be exaggerated if proxies underestimate HRQOL. However, in contrast to the situation in our previous validation study, in which patients and their proxies were interviewed within 72 hours of ICU admission, these investigators interviewed patients 3 months after ICU discharge, and their proxies at study entry. This makes it entirely possible that survivors of critical illness may overestimate pre-admission HRQOL.

A fourth limitation is that we only included patients with an ICU stay longer than 48 hours, because we aimed to evaluate in particular the sickest patients surviving critical illness. Clearly, this selection makes definite conclusions regarding HRQOL as a predictor of mortality impossible. Nevertheless, the combination of the APACHE II score with HRQOL scores improved the correct prediction of survival. A final potential limitation of the study is that this was a single centre study and the results may not be generalizable to other ICU populations with different patient populations or staffing situations.

## Conclusion

Pre-admission HRQOL, as estimated using a single-item question, in critically ill patients is as good at predicting survival/mortality as the APACHE II score. Initial evaluation of HRQOL can be done with the single-item question, because the SF-36 (PCS and MCS) yielded comparable results. The value in clinical practice of using the pre-admission HRQOL (PCS, MCS and general question) and the APACHE II score to provide useful predictive information in order to inform decision making appears to be limited, because of limitations in these models' abilities to predict survival/mortality in individual cases. Incorporating HRQOL into prediction models does not improve the predictive capacity of established models such as the APACHE II score. Nevertheless, it appears sensible to incorporate assessment of HRQOL into the many variables that may be considered when deciding whether a patient should be admitted to the ICU.

## Key messages

• Estimate of HRQOL before ICU admission is as good at predicting survival/mortality as the APACHE II score.

• The value of HRQOL measures and the APACHE II score is limited in clinical practice for making decisions in individual cases.

## Abbreviations

APACHE = Acute Physiology and Chronic Health Evaluation; AUC = area under the curve; HRQOL = health-related quality of life; ICU = intensive care unit; LASA = linear analogue self assessment; MCS = mental component score; PCS = physical component score.

## Competing interests

The authors declare that they have no competing interests.

## Authors' contributions

All authors contributed substantially to the study. JGMH analyzed and interpreted the data and drafted the manuscript. PES conceived of the study, contributed to the interpretation and analysis of the data, and revised the manuscript for important intellectual content. JHR conceived of the study, contributed to its design and the interpretation of the data, and revised the manuscript for important intellectual content. HFvS conceived of the study, contributed to the analysis and interpretation of the data, and revised the manuscript for important intellectual content. AJPS contributed to the interpretation of the data, and revised the manuscript for important intellectual content. JB contributed to the design and the interpretation of the data, and revised the manuscript for important intellectual content. All authors approved the final version submitted for publication.
